# Prospects of using strategic communication in sustainable tourism promotion

**DOI:** 10.3389/fspor.2025.1623121

**Published:** 2025-11-11

**Authors:** Odil Radjabov, Istamkhuja Olimovich Davronov, Mohichehra Boltayeva, Muborak Ashurova, Layli Navruz-zoda

**Affiliations:** Faculty of Economics and Tourism, Bukhara State University, Bukhara, Uzbekistan

**Keywords:** sustainable tourism principles, communication tools, sustainability indicators, strategic communication, communication efficiency

## Introduction

According to https://www.Booking.com surveys, 83% of travelers view sustainable travel as essential, with over two-thirds expecting more eco-friendly options from the tourism sector. More than half believe the COVID-19 pandemic has prompted them to adopt more sustainable habits. As a result, sustainability has become a critical concern for tourists, host destinations, and stakeholders. It encompasses public concerns related to water, air, cultural and natural heritage, and overall quality of life.

As outlined by United World tourism organization (UNWTO, currently UN Tourism) (1), key actors responsible for sustainable tourism include national and local tourism authorities, relevant ministries (e.g., Trade, Environment, Transport, Culture, Health), and mass media. Targets 12.8 & 13.3 (Call for public awareness, support with relevant information and education to promote sustainable consumption and production, and climate change) and Targets 16.6 & 16.10 (Require countries to establish accountable and transparent institutions to ensure public access to information) of Sustainable Development Goals (SDG) highlight the role of communication in sustainability achievement. Moreover, the 45 paragraph Agenda of SDG for 2030 (2). The significance of strategic communication in advancing sustainable tourism is demonstrated in multiple case studies (3):

- Kupang City (Indonesia) faced a primary barrier of low public awareness. Communication efforts were largely promotional, aiming to boost visibility. However, without complementary educational or participatory initiatives, long-term behavioural change was limited. Lesson: Awareness campaigns must be coupled with engagement strategies to sustain impact.
- Angkor (Cambodia) employed multi-stakeholder workshops to align conservation goals with tourism development. This participatory approach strengthened local buy-in and improved policy coherence. Best Practice: Facilitating dialogue among diverse stakeholders fosters ownership and shared responsibility for heritage protection.
- Georgetown (Malaysia) focused on adaptive reuse of cultural heritage buildings, integrating heritage storytelling into its branding. While this enriched visitor experiences, inconsistent messaging across channels reduced the overall effectiveness. Lesson: Consistent, multi-platform communication is critical for reinforcing destination identity.
- Vigan City (Philippines) implemented educational campaigns targeting both residents and visitors to promote respect for heritage sites. This led to measurable improvements in site preservation behaviour. Best Practice: Education-driven communication can directly influence pro-sustainability actions.
- Avebury (England) prioritised local community engagement in decision-making. This strategy enhanced trust and minimised resistance to tourism development. Lesson: Early and ongoing community involvement prevents conflict and strengthens sustainability outcomes.

Moreover, in case of Costa Rica strategic communication served as the main factor to development in tourism sector focusing on collaboration with stakeholders [especially with non-governmental organizations (NGOs)], multilingual activities and sustainability certification for stakeholders (4). These measures elevated the country's eco-tourism brand internationally and fostered strong public–private partnerships.

Recent studies largely concentrate on prevalent issues such as the preservation, use, and communication of cultural heritage within sustainability indicators as a technical issue. However, they often neglect the limited understanding among relevant authorities about the core principles of sustainable tourism in communication process. For instance, in practice the government-run websites frequently lack comprehensive or well-organized content on regional sustainable development. Instead, they typically highlight statistics related to accommodations or dining options, thereby relegating the cultural and touristic value of heritage sites to a secondary position, leaving a gap in sustainable tourism indicators communication through tourism sites. For example, cases with uninformed day-tripper fees in Venice, Italy (5) while there was insufficient collaboration with local community or in case of Komodo Island, Indonesia (6) where government didn't consult with stakeholders about closure, and subsequently all plans failed instead of approaching sustainability. This article examines how sustainability indicators can be integrated and effectively communicated through sustainable tourism practices. To achieve comprehensive results, the article addresses key topics including the dimensions and principles of sustainable tourism, phases of communication, communication tools for sustainability promotion, sustainability indicators related to communication and the role of strategic communication in sustainable tourism. The main goal article to integrate sustainable tourism indicators with communication efficiency indexes to balance technical and sustainability efficiency of strategic communication in case of sustainable tourism promotion.

## Methodology and sources

This study applies a two-phase approach combining a systematic literature review and content analysis.

### Phase 1 – Literature review

Academic sources (2010–2024) were retrieved from *Scopus, Web of Science,* and *Google Scholar* using terms such as “*sustainable tourism communication”, “*sustainability indicators” and “*tourism communication phases”*. Inclusion criteria: peer-reviewed studies or institutional reports relevant to sustainable tourism communication; exclusion criteria: non-academic sources, unrelated studies, duplicates. Screening involved title/abstract checks and full-text review. Relevant publications were analysed thematically to explore the integration between communication tools and communication phases (before, during, and after travel) in the context of sustainable tourism principles.

### Phase 2 – Content analysis

Six sustainability indicator systems where communication plays a significant role were purposively selected based on their global relevance, applicability to tourism destinations, and explicit inclusion of communication-related criteria:

- World Tourism Organization (1) Indicators of Sustainable Development for Tourism Destinations: A Guidebook
- Orientation-structure-Ergonomics-Content (OSEC) (7)
- Index General Communication Efficiency (IGEC) (8)
- Valencian Network of Smart destinations (SRDV) indicators (9)
- Self-assessment of sustainability based on SDG for tourism targets (10)
- The Global sustainable tourism council (2019) Destination Criteria version 2.0 for sustainable tourism destinations (GSTD) (11)

Findings were synthesized to compare how each system integrates communication into sustainability assessment, highlighting overlaps, unique elements, and best practices.

## Communication tools, phases and principles for sustainable tourism promotion

From the side of government and stakeholders of the tourist destination, UNWTO (12) emphasized several effective communication channels and interpretation tools for promotion of sustainable tourism:

- **Tourist Information Centres (TICs)**: government-supported facilities that provide direct, face-to-face guidance to visitors.
- **Signage and interpretation panels**: clearly designed and strategically located information aids.
- **Visitor Centres**: combine interpretive resources with service functions.
- **Accommodation providers**: serve as points for informing guests about local customs and resource conservation.
- Tour guides: play a key role in delivering interpretive content. In Argentina, local guides effectively used social media to raise awareness of regional wildlife and ecological values.
- **Holiday company representatives**: offer general orientation about destinations.
- **Local communities**: through education initiatives (schools, universities, workshops), residents can become informed advocates for sustainable tourism and engage in community-based tourism initiatives.

Spenceley and Rylance (13) offer practical guidelines for responsible tourism communication, recommending that stakeholders encourage visitors to follow sustainable practices at each stage of travel.

Before the visit: Tourists are advised to use online platforms to check for sustainability certifications (e.g., accommodations, transport, dining), learn basic local phrases, and research local public transport options.
During the visit: Visitors should support local businesses by purchasing eco-friendly souvenirs, follow resource-saving practices, diversify their spending across small enterprises, and respect local customs and community norms.
After the visit: Tourists can contribute by sharing their experiences via social media or word of mouth, posting photos, offering reviews, and supporting local initiatives through donations or charitable contributions.

When assessing tourism service providers, especially accommodations, Väisänen et al. (14). propose a four-dimensional sustainability framework as the focus on sustainability promotion in destinations:

Environmental sustainability: Efficient energy use, pollution reduction, noise control, maintenance, sustainable materials, and accessible transportation.
Social sustainability: Safety, healthy choices, use of local products, clear signage (25), services for diverse customer groups, local and youth employment, equality, and crowd control.
Cultural sustainability: Promotion of local traditions, cultural relevance, food heritage, and involving locals as cultural ambassadors.
Economic sustainability: Delivering value for money to consumers.

Considering these principles and tools, it becomes essential to develop and apply robust indicators for managing and evaluating strategic communication in sustainable tourism (15).

## Integration of sustainable tourism indicators and strategic communication issue

Strategic communication in case of development of cultural tourism, especially sites with rich heritage resources can be considered as an essential tool for promotion (8) and capacity building to awareness raising and heritage conservation. Teruel Serrano (16) defined strategic communication as the form where technology is utilized in order to accomplish sustainable tourism development by shared information access among authorities, stakeholders and residents, future through analyzing 120 cases regarding online communication means of protected areas (while they used communication strategically or spontaneously) she tried to create communication Efficiency questionary which potentially contributes development of General Index for communicative efficiency of Information and Communicational technologies (ICT) as main indicators for assessment.

Countries like Costa Rica and Slovenia aligned with Global Sustainable Tourism Council and implemented its indicators. Regarding strategic communication they used branding, collaboration, education, digital storytelling and sustainability certification activities to promote indicators such as cultural preservation, supporting local economy, destination planning, carbon footprint, waste management, visitor satisfaction and local community perceptions. However, communication has a wide range of opportunities to promote all indicators (4).

Since 1992, UNWTO (12) has progressively developed and refined indicators to support sustainable tourism, aiming to enhance decision-making, identify emerging challenges, evaluate impacts, monitor sustainability, and reduce risks. The organization proposed key criteria for selecting sustainability indicators, including relevance, feasibility, credibility, clarity, and comparability. Later, White et al. (17) expanded this list by adding attributes such as measurability, sensitivity, economic viability, acceptability, usability, reliability, participation, verifiability, replaceability, specificity, timeliness, transparency, and scientific grounding.

Altamirano et al. (18) proposed the Communication 2.0 Index for evaluating official tourism websites, focusing on key performance indicators (KPIs) related to their technical and interactive features. These include:

- **Growth**: measured by the increase in follower numbers.
- **Activity**: based on the frequency of content publications.
- **Service level**: assessed through analytic tools that evaluate user interactions.
- **Participation**: reflected by metrics such as likes, reactions, comments, and shares.
- **Engagement**: calculated as the percentage ratio of total interactions (likes, comments, posts) to the number of followers.

Complementarily, Baggio et al. (19) introduced the Website Quality Index (WQI), classifying website characteristics into six broad categories—first impression, design, content, structure, interactivity, and technical performance—along with five functional groups: informational content, customer relationship features, interactive services, Web 2.0 capabilities, and e-commerce functionalities.

However, the application of these indicators often differs between policy formulation and local implementation due to the unique characteristics of each destination (20, 21). Analyzing these differences can aid in addressing challenges like overtourism. Although indicators raise awareness of sustainability, governments may not always prioritize their implementation due to practical or political constraints (22, 23).

Table 1 illustrates comparative analysis of several authors sustainability indicators regarding communication issue:

**Table 1:** Indicators systems for SDG (sustainable development goals), STD (sustainable tourism development) and SC (sustainable communication).

#№	Organization or research	Focus	Total Number of criteria, issue and indicator	Communication issues	Variables regarding communication
1	World Tourism Organization (1) Indicators of Sustainable Development for Tourism Destinations: A Guidebook	STD	Total 768 indicators, 12 baseline issues and 29 basic indicators	- Tourist satisfaction - Local community perception - Marketing	- Local satisfaction with tourism - Sustaining tourist satisfaction - Educational-interpretive value: - Awareness raising - Local community participation (courses, meetings, promotion of content in the curriculum of local educational system, etc.) - Visitor and intermediaries’ satisfaction
2	Orientation-structure-Ergonomics-Content (OSEC) (7)	SC	4 dimensions with 19 subdimensions	64 variables	All 64 variables - Orinetation (6 variables) - Structure (17 variables) - Ergonomics (19 variables) - Content (22 variables)
3	Index General Communication Efficiency (IGEC) (8)	SC	3 Criteria, 11 indicators and 33 variables	3 Criteria, 11 indicators and 36 variables	All 11 indicator and 36 variables - Tourism–Heritage Relations (23 variables) - Tourism Training (3 variable) - Strategic communication (11 variables)
4	Valencian Network of Smart destinations (SRDV) indicators (9)	STD	9 Sections, 72 indicators	Section 1: Governance Section 2: Sustainability Section 3: Accessibility Section 5: Connectivity Section 7: Information system Section 8: Online marketing	1.9 Social awareness campaigns for citizens 1.10 Application for tourism initiatives 2.3. Public promotion of sustainable mobility 2.8 and 14 Development of awareness campaigns for STD 3.2 Accessible information for disabilities 3.3. Web Accessibility Initiative (WAI) 5. Total technical facilities of connect 7. Total ICT technologies 8. Total marketing
5	Self-assessment of sustainability based on SDG for tourism targets (10)	SDG	17 SDG, 61 variables	- SDG8 - SDG9 - SDG12 - SDG14/15 - SDG16 - SDG17	8.4. Technological innovation 9.3 Use of ICT for ST 12.4 Collaboration between sending and receiving regions 14.3,4/15.4 Awareness raising and promotional actions for land and water resources 16.1 Citizen participant 16.3 Resident and visitor satisfaction 16.4 Information of Tourist interest 17.2 Exchange of good practise 17.3 Co-operation for innovation in sustainable development
6	The Global sustainable tourism council (2019) Destination Criteria version 2.0 for sustainable tourism destinations (GSTD) (11)	STD	4 main section of criteria (10 subsection) with 38 indicators and 174 variables	- A2 Destination management strategy and action plan - A4 Enterprise engagement and sustainability standards - A5 Resident engagement and feedback - A6 Visitor engagement and feedback - A7 Promotion and information - B5 Preventing exploitation and discrimination - B8 Access for all - C3 Intangible heritage - C7 Site interpretation - D2 Visitor management at natural sites	- A2c. Evidence of stakeholder consultation, meetings etc. in developing the plan. - A4a. Evidence of regular communication - A4b. Sustainability support and advice to tourism related business - A5 Residents engagement and awareness rising; - A6 Visitor satisfaction surveys - A7 Current information and promotional material - B5 Information on accessibility included in communications about the destination as a whole. - B8 Examples of celebration and visitor experiences of intangible cultural heritage (events, distinctive products etc.). - C3. Visitor feedback, - C7 Interpretative information, pre-arrival information - D2 Provision of training for guides.

Source: own elaboration based on the research of other authors (1, 7–11).

According to Table 1, the sustainability indicators most closely related to communication primarily focus on tourist and local community satisfaction, levels of awareness, and the promotion of sustainable practices (3). Additionally, some indicators address the technical aspects of communication, such as the effectiveness of digital platforms, information dissemination tools, and other innovations (24).

Although Table 1 presents a diverse range of sustainability indicator systems in tourism, a strategic communication perspective reveals a lack of consistent depth in how these frameworks address communication as a tool for sustainability. For example, the SDG-based self-assessment tool incorporates 61 variables, several of which (e.g., SDG17.17, SDG16.3) highlight collaboration, citizen engagement, and transparent governance. However, it does not fully operationalize how communication strategies can foster stakeholder alignment or behavioral change. In contrast, the UNWTO framework emphasizes community perception and tourist satisfaction but approaches communication more as a metric of reception rather than a proactive strategy for engagement or policy diffusion.

The SRDV framework takes a more structured approach to communication, explicitly integrating online marketing, information systems, and web accessibility under the smart destination model. This aligns better with the principles of strategic communication by acknowledging the role of digital tools in shaping narratives, building awareness, and promoting participatory governance. However, its focus is somewhat technocratic, emphasizing tools over message framing or audience segmentation.

The OSEC and IGEC models focus almost exclusively on communication variables, offering 64 and 36 variables respectively, and are closer to frameworks that can inform strategic communication planning. Yet, they remain largely internal or operational in scope and do not connect communication performance directly to sustainability outcomes. This fragmentation across frameworks suggests a conceptual gap: while communication is widely recognized, its strategic role in influencing sustainability behavior, managing perceptions, and ensuring multilevel governance coherence remains underdeveloped.

The UNWTO, SRDV, SDG Self-assessment, and GSTD systems primarily treat communication as a technical component, focusing on aspects such as accessibility, transparency, and stakeholder feedback. In contrast, the IGEC and OSEC systems are fully dedicated to communication, addressing both its technical dimensions and its role in sustainability.

OSEC, IGEC, and SRDV treat communication as a primary focus, offering high strategic value for destination branding, organizational messaging, and digital platforms. UNWTO, SDG Self-assessment, and GSTD include communication indirectly, focusing on areas like awareness, stakeholder engagement, and accessibility. Strategic value is highest when communication is central (OSEC, IGEC, SRDV), and lower when it is supportive.

**Figure 1:**
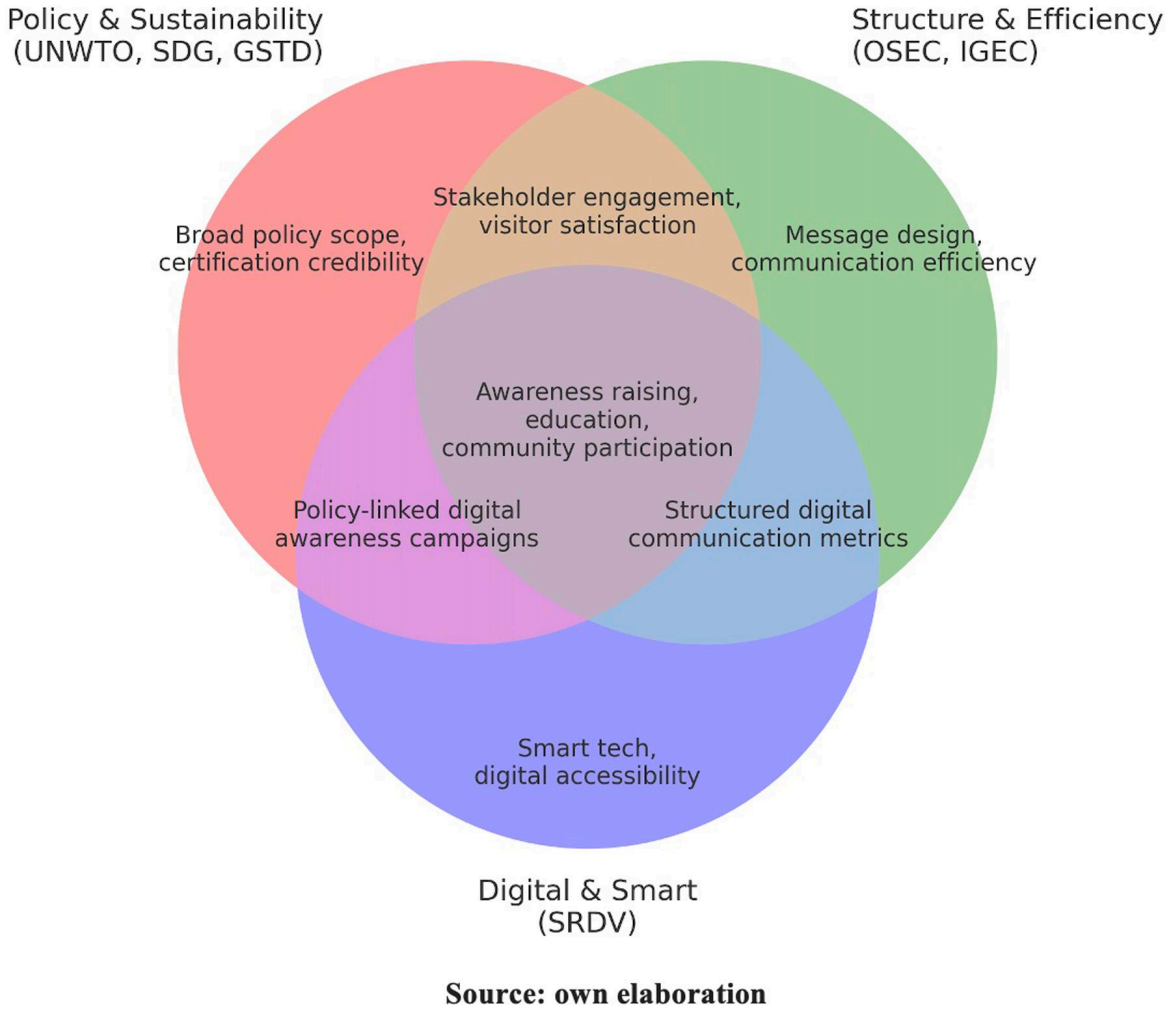
Integration of tourism communication indicator frameworks. Source: own elaboration.

The Figure 1 groups the six analysed frameworks into three thematic clusters: Policy and Sustainability (UNWTO, SDG Self-Assessment, GSTD), Structure and Efficiency (OSEC, IGEC), and Digital and Smart (SRDV). The unique sections highlight each group's strengths—policy scope and certification credibility; message design and communication efficiency; smart technology and digital accessibility. Overlaps represent shared priorities:

- Policy and Structure: Stakeholder engagement and visitor satisfaction.
- Policy and Digital: Policy-linked digital awareness campaigns.
- Structure and Digital: Structured digital communication metrics.

All Three: Awareness raising, education, and community participation.

This integrated view clarifies how frameworks complement one another and guides destinations in selecting or combining them according to development stage, technological capacity, and sustainability objectives.

## Conclusion

Strategic communication plays a critical role in promoting sustainable cultural tourism. Digital tools such as websites, social media, and mobile platforms enable transparent communication, engage visitors, and encourage responsible behavior. However, destination management organizations (DMOs) need to place greater emphasis on developing tools for awareness raising—including training programs, cultural performances, community meetings, and collaborative initiatives. Key indicators—such as tourist and local community satisfaction, awareness levels, and the promotion of sustainability practices—help measure the effectiveness of communication strategies. Furthermore, these indicators serve as essential outputs that inform DMOs' future communication planning and actions.

Tourism communication frameworks vary in focus and application. To enhance strategic communication, stakeholders should apply the most relevant systems:

1. Local Governments

Goal: Ensure sustainability policies are visible, accessible, and inclusive.

Recommended Frameworks: UNWTO, SDG Self-Assessment, GSTD.

Actions:

- Integrate sustainability objectives into tourism master plans.
- Establish monthly community forums for feedback and co-planning.
- Ensure all promotional materials are available in multiple languages.

Toolkit Examples:

- *Policy Brief Template* for public dissemination of sustainability goals.
- ***Event Sustainability Checklist* for cultural festivals.**

1. Destination Management Organizations (DMOs) & Tourism Planners

Goal: Improve branding, communication efficiency, and digital outreach.

Recommended Frameworks: OSEC, IGEC, SRDV.

Actions:

- Develop a content calendar for sustainability-focused social media campaigns.
- Use SRDV tools to implement interactive digital maps and visitor feedback systems.
- Conduct staff training on effective sustainability messaging.

Toolkit Examples:

- Sustainability Campaign Template (visual + caption guidelines).
- Training Module Outline for sustainable tourism communication.

1. Community Groups and Cultural Stakeholders

Goal: Engage in co-creating culturally relevant sustainability messages.

Recommended Frameworks: UNWTO, GSTD.

Actions:

- Nominate community ambassadors to communicate sustainability practices to visitors.
- Organise storytelling events showcasing heritage preservation success stories.
- Create visual guides (infographics/posters) for sustainable visitor behaviour.

Toolkit Examples:

- *Poster Template* for heritage respect, waste reduction, and local product promotion.
- *Volunteer Handbook* outlining roles, responsibilities, and incentives.

Ultimately, the choice of communication indicator system depends on the specific goals and needs of the destination. By aligning frameworks with stakeholder roles, tourism destinations can strengthen communication, foster inclusivity, and support sustainable development. Moreover, communication indicators should be directly integrated into sustainability assessment systems to ensure they receive appropriate significance in tourism planning.
